# Laser-induced graphene trending in biosensors: understanding electrode shelf-life of this highly porous material

**DOI:** 10.1007/s00216-023-05082-y

**Published:** 2023-12-12

**Authors:** Arne Behrent, Veronika Borggraefe, Antje J. Baeumner

**Affiliations:** Institute of Analytical Chemistry, Chemo- and Biosensors, University of Regensburg, Universitätsstraße 31, 93053 Regensburg, Germany

**Keywords:** Laser-induced graphene, Electrochemical biosensor, Reproducibility, Shelf-life, Stability, Morphology

## Abstract

Laser-induced graphene (LIG) has received much attention in recent years as a possible transducer material for electroanalytical sensors. Its simplicity of fabrication and good electrochemical performance are typically highlighted. However, we found that unmodified and untreated LIG electrodes had a limited shelf-life for certain electroanalytical applications, likely due to the adsorption of adventitious hydrocarbons from the storage environment. Electrode responses did not change immediately after exposure to ambient conditions but over longer periods of time, probably due to the immense specific surface area of the LIG material. LIG shelf-life is seldomly discussed prominently in the literature, yet overall trends for solutions to this challenge can be identified. Such findings from the literature regarding the long-term storage stability of LIG electrodes, pure and modified, are discussed here along with explanations for likely protective mechanisms. Specifically, applying a protective coating on LIG electrodes after manufacture is possibly the easiest method to preserve electrode functionality and should be identified as a trend for well-performing LIG electrodes in the future. Furthermore, suggested influences of the accompanying LIG microstructure/morphology on electrode characteristics are evaluated.

**Supplementary Information:**

The online version contains supplementary material available at 10.1007/s00216-023-05082-y.

## Introduction

There is a need for localized chemical and biological testing in the world to name the monitoring of the natural environment for accidentally or intentionally released substances, early identification of outbreaks of infectious diseases to prevent a pandemic, and monitoring of chronic illnesses to provide continuous or highly frequent health assessments and hence improve a patient’s life as three highly relevant examples. Thus, quick, low-cost point-of-care sensing is needed to support the envisioned and anticipated shift of diagnostic testing toward on-site and personalized monitoring to improve medical care. The same technologies are and will be applied to smart farming, environmental monitoring, food safety control, and the control of industrial production processes. Electrochemical sensors are a suitable tool to fulfill those needs due to their high robustness, low operation cost, and small footprint.

Laser-induced graphene (LIG) is a relatively new porous graphitic material, which has been adopted by many electroanalytical sensor research groups mostly due to its quick, adaptable, and easy route of fabrication as nicely demonstrated in several recent reviews [1–3]. LIG is made by rapidly heating a carbon-containing substrate with a strong focused laser, causing the substrate to partially evaporate and reform into few-layer graphene sheets that are randomly arranged in a porous structure. Macroscopic patterning is achieved by guiding the laser spot with a motion control system, e.g., a commercial laser-cutter instrument connected to a computer. Although many different substrates [4–7] and lasers [8–10] have been demonstrated as amenable to this method, the most common basis remains a thin sheet of polyimide processed by a CO_2_ laser, as reported in the original publication [11] by James Tour’s group in 2014, who have also reviewed key aspects of their LIG-related research [12]. LIG is very useful for prototyping, because patterns can be modified quickly and porosity and hydrophilicity of the material are to a certain degree tunable through adjustment of the lasing parameters [13–17]. It has been suggested before that LIG electrodes can be mass produced by roll-to-roll fabrication [12] and the reported signal variation between electrodes of the same batch and between different batches seems appropriately low [16] to be confident in using this method for commercial production.

A major requirement for widespread application of single-use LIG electrodes is a stable electrochemical response over extended periods of time. Considering the reactive nature of the material and its high surface area, it is expected that environmental conditions during storage influence the chemical and physical characteristics of the material. Thus, not surprisingly, the hydrophilicity of the electrode and capacitive currents in cyclic voltammetry can be observed to decline when electrodes of the same batch are stored for extended periods of time. This effect may vary significantly depending on the analyte species, electrode storage conditions, and electrode modification. This was, for example, experienced in our lab when quantifying acetaminophen where signal variation between electrodes stored for a few weeks was too high to create reliable calibration plots when employing multiple electrodes.

Surprisingly, aside from the hundreds of reports on LIG electrodes for chemical sensing or biosensing, their long-term signal stability is seldomly addressed albeit it should be a well-known challenge. For example, in their very readable paper about affordable equipment to fabricate LIG electrodes, Costa et al. only sparsely report on LIG storage stability, by mentioning that the electrodes still showed the typical reversible CV profile of [Fe(CN)_6_]^4−/3−^ after being stored for 1 year at room temperature [18]. Another publication mentioned that LIG has reliable environmental stability, although just casually and without further reference [19]. In contrast, the group of Wen Liaoyong recently mentioned explicitly that “Laser-induced graphene […] suffers from serious decay in long-term biosensing, which greatly restricts its practical applications.” [20]. Parts of this apparent disagreement on signal stability could be based on the fact that materials with different properties are summarized under the same term “laser-induced graphene,” which is defined by its method of fabrication. Since a host of factors influence the fabrication method, it offers a broad spectrum of porous carbon materials [16]. Of course, any fabrication process is subject to variations, but with what is termed LIG, this case is especially strong. A second explanation for the perceived underreporting on LIG stability could be that some measurement techniques and analytes are not very sensitive to changes in electrode surface characteristics, e.g., when outer-sphere redox species are employed as redox probes in a sensing scheme. Moreover, when intentional modifications are made to the electrode surface—as in the case of, e.g., deposited polymer films or nanoparticles, immobilized biomolecules, or coated membranes—the LIG surface is protected and less prone to any ageing effects during storage.

In this present report, we aim to highlight the existing issues with long-term storage of LIG electrodes, presenting some of our recent results, and what, if any, issues are addressed in the literature. The discussion is divided into three sections, starting with the response stability of simple unmodified LIG electrodes, including some of the recent findings from our lab, followed by an overview on the stabilizing effect of surface modifications as found in the literature. It ends with a brief discussion on the different LIG morphologies and microstructures found in literature and how they may affect sensor performance.

## Shelf-life of unmodified electrodes

Already in 2013, Li et al. had demonstrated via contact angle measurements and XPS that freshly exposed surfaces of graphene and graphite are more hydrophilic than traditionally thought [21]. Upon exposure to air, however, the surface quickly becomes less hydrophilic due to contamination with ever-present hydrocarbons on a timescale of minutes to hours, which was confirmed by others later [22]. The group of Patrick Unwin connected hydrocarbon contamination of highly ordered pyrolytic graphite to decreasing kinetics of the inner-sphere redox couple Fe^3+/2+^, a finding which matches with their new understanding that freshly cut basal-plane graphite is actually quite reactive [23].

Since LIG constitutes a spectrum of partially graphitic/graphenic surfaces mixed with regions of amorphous carbon, it is highly likely that ambient hydrocarbons contaminate also this surface. Due to its high porosity and thus very large specific surface area, it seems feasible that it takes much longer to reach adsorption equilibrium on LIG than on regular graphite electrodes, which would explain the observed longer timescale of days or weeks, during which LIG displays changes in surface characteristics.

**Fig. 1:**
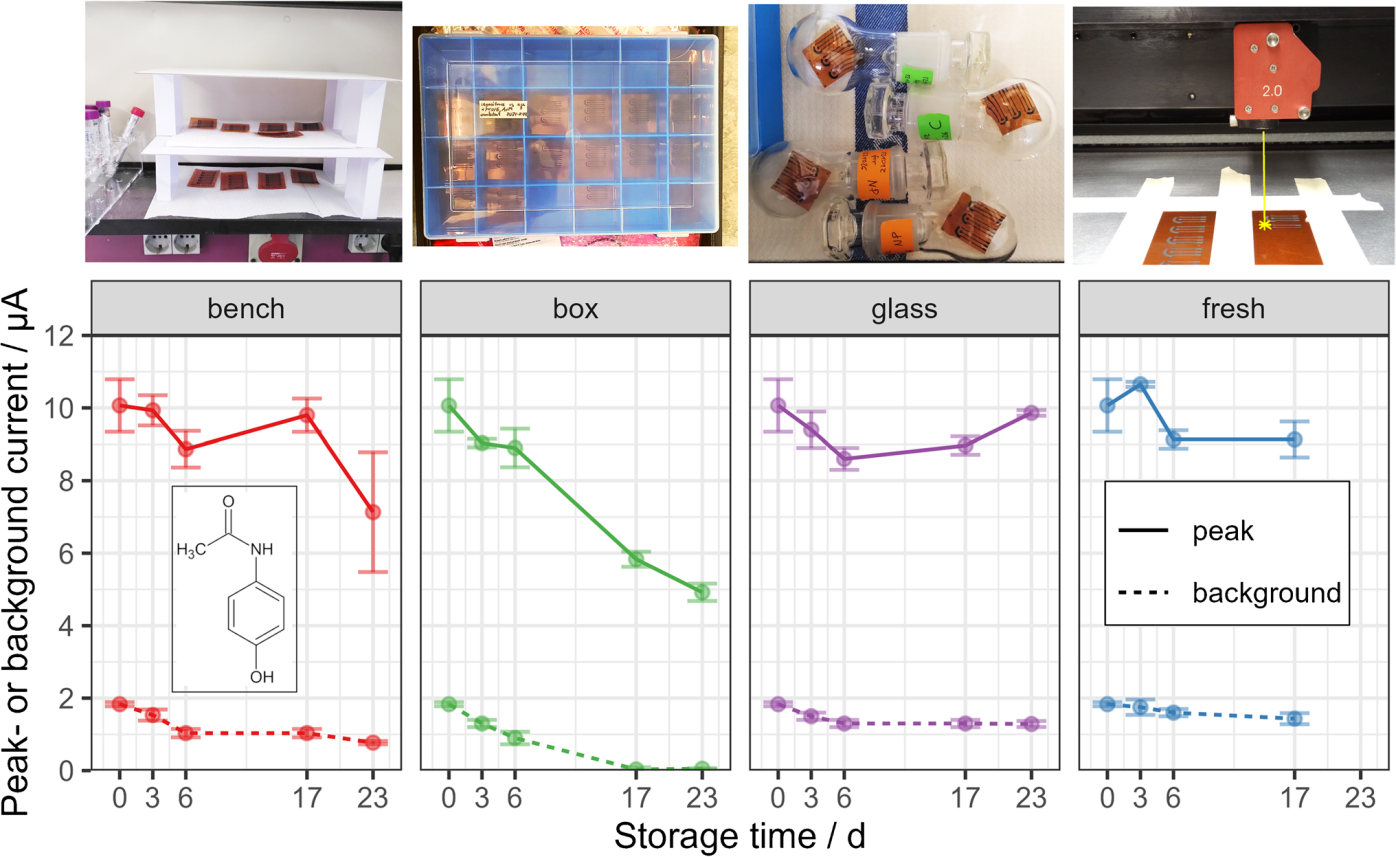
Oxidation peak current and background current from cyclic voltammetry of 0.1 mM acetaminophen vs. electrode storage time in different environments: on a lab bench, in a polypropylene box, or in glass flasks. Fresh electrodes were also prepared at each timepoint and tested alongside stored electrodes (error bars indicate 1 × SD, *n* = 3)

We systematically examined the effect of the storage environment on voltammetric response, using acetaminophen (APAP) as a probe. Details on the fabrication process and other experimental methods are described in the online resources. Temperature and relative humidity were not controlled in the storage environments, since they were assumed to be not significant for the outcome of voltammetric measurements. All fabricated LIG were used directly, i.e., none was discarded before measurement, and no results were excluded from the graphs. Figure 1 compares signals from electrodes stored over 2–3 weeks either openly on a lab bench, in a polypropylene box, or in glass flasks (without grease). The faradaic and capacitive currents of electrodes in the plastic box dropped significantly by 51% [*t*(2.57) = 11.41, *p* = 0.003] and 97% [*t*(2.41) = 50.7, *p* < 0.001], respectively, when comparing first vs. last set of values in the experiment. No statistically sound trend regarding faradaic currents was detectable with electrodes stored in glass flasks (*p* = 0.67) and on the bench (*p* = 0.21), although the capacitive current of bench samples also dropped by 58% [*t*(2.73) = 22.9, *p* < 0.001]. The variation between daily prepared control electrodes demonstrated an underlying issue with overall reproducibility. However, peak currents did not decline accordingly when [Ru(NH_3_)_6_]^3+^ or [Fe(CN)_6_]^4−^ were employed as redox probes (see Fig. S 5 in the online resources). This makes sense, since [Ru(NH_3_)_6_]^3+^ exhibits perfect outer-sphere electron transfer and thus should not be affected by a thin surface film. While the electrochemical behavior of [Fe(CN)_6_]^4−^ on carbon is probably complicated [24], it is still less susceptible to surface contamination than APAP, which undergoes adsorption for electron transfer [25].

**Fig. 2:**
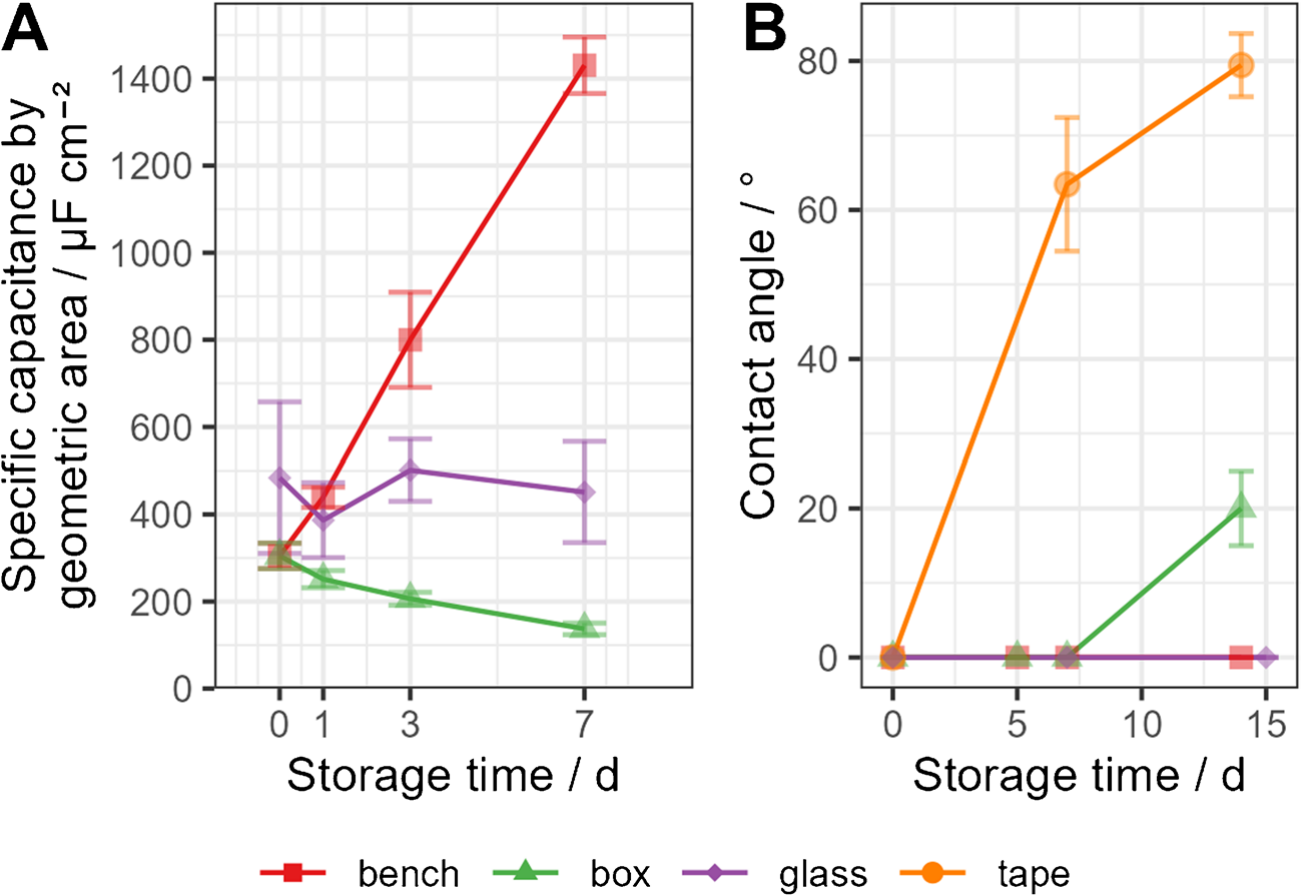
LIG surface capacitance (**A**) and water contact angle (**B**) over time in different storage environments without nail polish. Capacitance of “glass” group obtained in a separate experiment. Error bars represent the single standard deviation of the sample. Sample size was *n* = 5 for capacitance measurements and for contact angle on “tape” samples. Surface wetting was perfect (angle = 0°) in all other cases except for “box” samples after 14 days, which allowed measurement of a transient value, with roughly estimated error

Trends for electrode capacitance (measured via CV in normal phosphate-buffered saline (PBS)) and static water contact angles on LIG are shown in Fig. 2. Samples stored in the plastic box lost 55% of their initial capacitance within 7 days [*t*(5.58) =  − 11.7, *p* < 0.001] and their surface became slightly less hydrophilic after 14 days. This observation supports the hydrocarbon contamination hypothesis since a slow release of volatile organic compounds from walls and lid of the container (both made of PP) is plausible. LIG samples stored on the bench remained hydrophilic with a contact angle of 0° over 2 weeks of observation. Interestingly, Wen’s group reported that water contact angles on unmodified LIG, stored under ambient conditions, increased to 141° within 15 days, but the material already featured a contact angle of 70° on the day of fabrication, due to the set process parameters [20]. This might indicate that already more hydrophobic surfaces increase their water contact angles quicker under otherwise similar contamination scenarios. More prominently, instead of dropping in its value, the capacitance of bench-stored electrodes increased roughly fourfold within a week [+ 369%,* t*(5.57) = 35.4, *p* < 0.001]. This rising capacitance cannot be explained by a lack of contaminating hydrocarbons alone and could be a product of surface oxidation through atmospheric dioxygen [24]. Lastly, the capacitance of samples stored in glass flasks displayed no trend (*p* = 0.71), indicating that the pristine surface of those samples was better preserved over the course of the study. Some LIG samples designated for contact angle measurements were fixed onto a glass slide with a double-sided adhesive tape and stored in a drawer (see Fig. S 1 in the online resources). The tape seems to have strongly emitted contaminants which deposited onto the sample surface, as indicated by the quick rise in water contact angle (Fig. 2B).

**Fig. 3:**
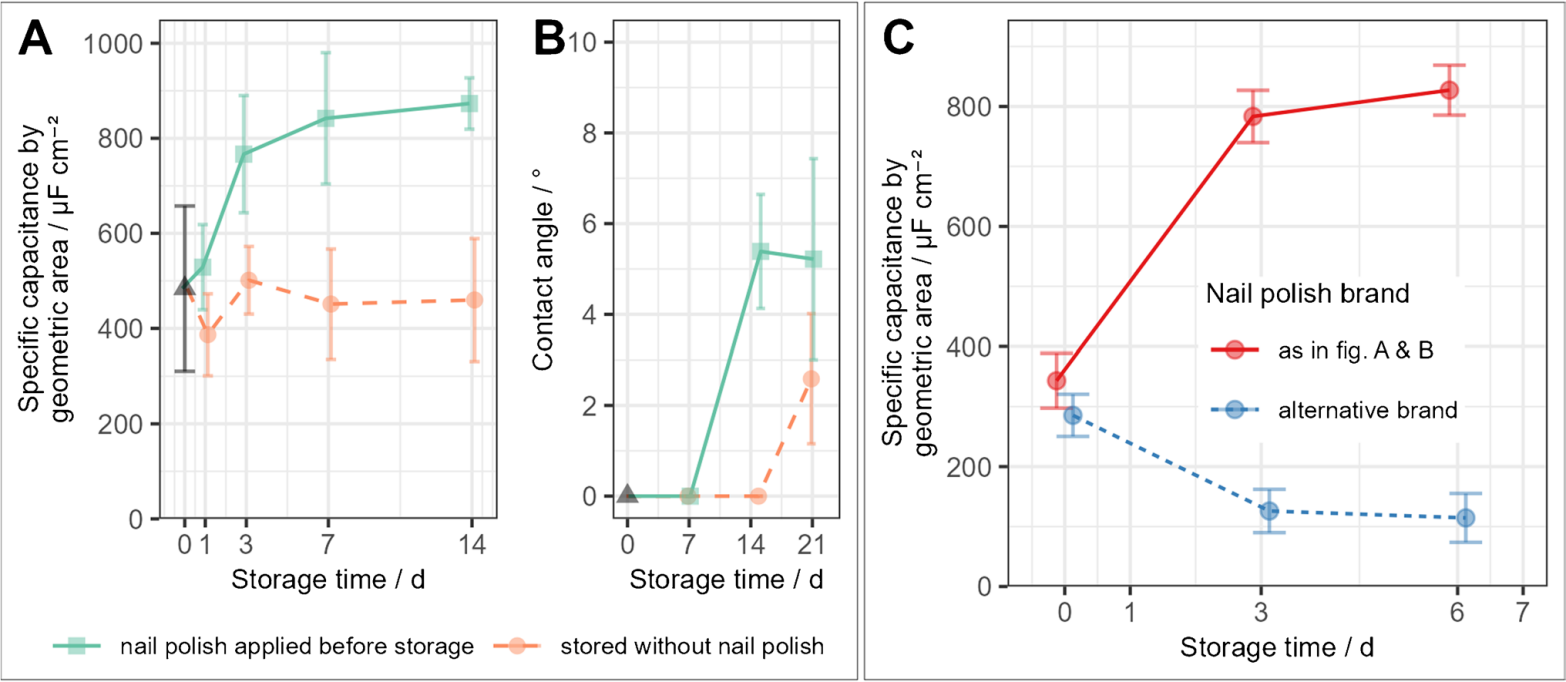
Effect of nail polish application during storage on LIG kept in glass flasks: **A** capacitance (*n* = 6), **B** contact angle (*n* = 3), **C** different effects of two nail-polish types on capacitance (solid line = “Catrice,” dashed line = “essence,” *n* = 6)

Since the tape was identified as a source of hydrocarbon contamination, we also evaluated the nail polish, which we customarily use to insulate current collectors on the LIG electrodes. Nail polish was applied to samples and allowed to dry for 30 min before storage in glass flasks. A scheme of the electrode geometry with nail polish-covered area is shown in Fig. S 1. Surprisingly, the capacitance of electrodes stored with nail polish increased significantly during the first week, while electrodes kept without nail polish remained overall unchanged (Fig. 3A). A tiny shift toward a more hydrophobic surface was observed after 14 days. However, the contact angles in Fig. 3B were all transiently extracted from droplets, which underwent perfect spreading within 5 s or less. The presence of nail polish during storage thus only minimally affected water contact angles, much less than the results with sticky tape, displayed in Fig. 2C. Different brands of seemingly identical commercial nail polish were incidentally found to have opposite effects on electrode capacitance over time (Fig. 3C). Both brands were simple nitrocellulose-based varnishes having the same appearance and containing a similar mix of solvents but varying in additives (see Tab. S 1 in the online resources).

To summarize our findings on unmodified LIG electrodes, pristine surfaces are likely prone to adsorb hydrocarbon contaminants from the storage environment, which depending on the concentration in that environment may strongly affect surface wettability and analyte signals in voltammetry. Adsorbing analytes are likely most affected while the signal from outer-sphere redox species is stable over time. If a varnish is used for insulation, that may also be a source of contamination during long-term storage. A self-made varnish with a minimal list of ingredients (e.g., nitrocellulose + ethyl acetate) would strategically be preferable to a commercial product to avoid unknown interferents and possible supply chain issues. LIG surfaces also seem to oxidize on contact with air and light, which may be beneficial, depending on redox species.

The storage stability of untreated LIG was also discussed in the following reports, some of which also report beneficial surface modifications:

Matias et al. used unmodified LIG electrodes in a 3D-printed electrochemical cell for the detection of atropine via differential pulse voltammetry (DPV) and noted that the relative standard deviation from 20 µM of substance was only 4.7% for daily measurements on 10 days [26]. This is a reported case where unmodified LIG electrodes did not significantly degrade during storage. Unfortunately, the change of signal over time is not observable from the data which makes finding a trend difficult.

In a very thorough and readable publication on paper-based laser-pyrolyzed electrofluidics, Bezinge et al. describe the fabrication and characterization of paper-based LIG electrodes integrated into wax channels for fluid control [27]. The electroactive area (EA), measured via CV scans with [Fe(CN)_6_]^3−/4−^, was chosen as key index for storage stability. EA of untreated electrodes, stored at room temperature with desiccant, dropped to 40% on day 7 and to roughly 20% after 4 weeks, which is explainable through adsorption of contaminants. Another set of electrodes, whose wettability was increased initially with O_2_ plasma treatment, exhibited a less sharp decline of EA and retained roughly 70% after 4 weeks. The authors proposed that the treatment had irreversibly hydrophilized the surface.

The group of Liaoyong Wen thoroughly studied the storage stability of LIG under ambient conditions, measured as CV response to the [Fe(CN)_6_]^3−/4−^ couple, in two different modes: comparing electrodes of the same batch, which are discarded after a single measurement (singe-use scenario), or comparing the signal of the same set of electrodes, which are used repeatedly on several days (repeated-use scenario) [20]. Unmodified LIG electrodes of the same batch dropped to 50% signal after 5 days and 10% signal after 15 days in single use, while repeated use was barely possible, with the peak current already dropping to 67% on day 2. The authors tentatively explained this signal decline through the loss of oxygen-rich groups during storage, which seems unlikely. In order to increase signal stability of the desired uric acid sensor for continuous on-body sweat measurement, clusters of gold were electrodeposited and a coat of chitosan was applied. The authors suggest that Au clusters improve signal stability by covering surface defects in LIG, and in fact, the single-use signal stability of Au-modified electrodes without chitosan cover improved significantly, although the reusability was as low as for unmodified LIG. When a chitosan film was added, the signal stability was visibly improved, with single-use electrodes retaining 97% of initial peak current after 30 days of storage and repeatedly used electrodes exhibited 95% of signal after 10 days. Aside remarkably improving signal stability, the chitosan cover also significantly increased wettability, causing the absolute signal to almost double. It is likely that the chitosan film alone would already improve electrode stability for this application, although the gold nanoparticles seemed to stabilize the response, too. A chitosan-only control is present in some of the presented data, but not in large enough scope to draw conclusions.

## Shelf-life of LIG with surface modifications

In the previous section, we have assessed the storage stability of unmodified LIG electrodes. However, the vast majority of reported electrochemical sensors rely on further surface modifications, e.g., to achieve analyte specificity (DNA hybridization, immunosensors, molecularly imprinted polymers, etc.), or to facilitate signal generation from non-electroactive analytes (glucose).

Due to the obvious importance of body glucose monitoring, many examples of LIG-based glucose sensors have been published. Tehrani and Bavarian deposited cubic copper nanoparticles onto LIG to make an amperometric glucose sensor with a lower limit of detection (LOD) of 0.25 µM and a linear range from 0.025 to 4.5 mM [8]. Signal stability was tested by re-evaluating the same set of electrodes, which were stored in a petri dish under ambient conditions, in 3-day intervals with 0.1 mM glucose. The signal decreased on average only by 1.5% within 30 days, which is excellent. The lab of Xu Rongqing has recently published two reports on LIG-based non-enzymatic glucose sensors working in alkaline solutions: in one instance, the LIG surface was connected to a zinc foil and immersed into a solution of CuSO_4_ to deposit copper nanoparticles without the need for a power source [28]. The sensor was used for amperometry in stirred solution and exhibited a linear range of 1 µM to 6 mM and LOD of 0.39 µM. The sensor response only decreased by 5.96% within 3 weeks, when the same sensor was stored at ambient conditions and tested once every 3 days with 1 mM glucose. The reproducibility was considered high with a relative standard deviation of 2.7% between six electrodes. Very similar values for reproducibility (3.5%, *n* = 6) and stability (5.5% decrease) were reported in a separate account on a Co_3_O_4_ nanoparticle-based sensor [19]. Here, the authors dissolved cobalt chloride in liquid polyimide precursor to facilitate the formation of evenly distributed nanoparticles during laser scribing. Settu et al. immobilized glucose oxidase in a chitosan matrix on LIG without any further modifications [29]. They chose chronoamperometry over a stirred cell, which is more favorable for a point-of-care sensor, and recorded a current at 0.8 V, probably due to direct oxidation of H_2_O_2_ at the carbon surface. The sensor response to 5 mM glucose dropped to 90% of the initial value within the first 10 days of storage at 4 °C and further to 72–85% within 25 days. Lu et al. went a step further and combined glucose oxidase with previously deposited Pt nanoparticles as catalysts for H_2_O_2_ oxidation, which allowed recording at a lower potential [30]. Although a layer of nafion was applied to minimize enzyme loss, the signal decreased by approx. 25% over 2 weeks, during which the same sensor was tested once daily with 0.5 mM glucose.

All of the listed examples of non-enzymatic glucose sensors exhibited fairly stable signals when stored over a few weeks, while enzymatic sensors deteriorated faster. Adsorption of adventitious hydrocarbons onto LIG surfaces likely happened on all described non-enzymatic sensors, but had no significant effect on signal strength over time. Assuming that electron transfer happened only at the catalytic nanoparticles, and assuming further that these are unlikely to adsorb contaminants during storage, then LIG-based non-enzymatic glucose sensors are stable for that reason. Since the given examples of enzymatic sensors all involved a protective membrane (chitosan or nafion), it can be assumed that these did not accumulate hydrocarbons during storage but that rather enzyme degradation over time is the likely culprit for declining signals.

LIG-based sensors have been constructed for the detection of heavy metals by anodic stripping voltammetry. Although Pb^2+^ ions have been detected on unmodified LIG with limited success [31], most reported sensors make use of one or multiple additional modifications to promote heavy metal deposition. To fabricate a flexible sensor for copper detection in sweat, Hui et al. transferred LIG from PI onto poly-dimethylsiloxane (PDMS) through the infusion/peel-off method and then drop cast siloxene and carbon nanotubes for synergistic effects [32]. Although the relevance of routinely measuring Cu ions in sweat may be questionable, the authors ingeniously used an in-built pH sensor to compensate for impaired Cu ion detection at higher pH levels. In a stability study, the signal for 80 ppb of Cu ions dropped by 2.5% in 5 days. Repeatability and reproducibility were also good. Huang et al. made a composite electrode material using porous LIG with MoS_2_ sheets as a base followed by deposition of CeO_2_ and Au NPs in order to detect Cu and Zn ions in aqueous solution via stripping voltammetry [33]. The authors investigated the lifetime of sensors over the course of 5 weeks storage at room temperature and found that the signal from either analyte decreased less than 5% over the first 3 weeks and about 15% after 5 weeks, when probing with a concentration of 100 ppb. In another report, Jeong et al. obtained a sensor with excellent characteristics (LOD of 0.1 ppb and linearity up to 120 ppb for Cd, Pb, and Cu ions) simply by depositing silver nanoparticles onto LIG [34]. The electrodes, destined for use in drinking water, yielded stable signals from a mix of Cd, Pb, and Cu (each 200 ppb) over the whole testing period of 5 weeks. Unfortunately, it is not mentioned whether the same electrodes were measured repeatedly or how they were stored. Interestingly, the unmodified LIG control in this report also exhibited well-resolved stripping peaks for Cd and Pb. Lu et al. [35] combined electrochemically deposited poly-l-cysteine with a coating of ionic liquid to promote Pb^2+^ sensing on LIG, resulting in excellent LOD of 0.17 ppb and linear range of 1–180 ppb. The sensor, stored at 4 °C and tested weekly, retained 95.3% sensitivity after 4 weeks (*n* = 1). In all examples, the LIG-based heavy metal sensors exhibited a long shelf-life, possibly due to the detection mechanism involving metal or metal oxide nanostructures or an applied coating.

Finally, we close this section with a selection of sensors for relevant, other types of analytes: Bahamon-Pinzon et al. created an affordable amperometric sensor for organophosphorus pesticide monitoring through deposition of copper nanoparticles on LIG. Over the course of 21 days, the authors found that the signal intensity was independent of storage time but remarked that sensor reproducibility was low (with *n* = 3) and that improvements in the manufacturing process were necessary [36]. To create a sensor for the antibiotic and environmental pollutant chloramphenicol, Chang et al. decorated an LIG electrode with TiO_2_ nanoparticles dispersed in carboxymethylcellulose and also added silver nanoparticles [37]. The modification allowed direct analyte detection via DPV with a linear range of 0.01 to 100 µM. When the same electrode was tested once a day for 10 consecutive days, the signal dropped to 87.2% of the initial value, which is interpreted as good stability. The group of Jonathan Claussen has published several reports on ion-selective electrodes (ISE) made from LIG covered with PVC-based membranes. In one report about sensors for quantifying ions in human urine, they mention a stable electrode signal after 3 months dry storage at room temperature in which 100% and 86% of sensitivity remained for K^+^ and NH_4_^+^ respectively [38]. In other publications, the group reported excellent shelf-life of over 40 days for a pH sensor [39] and 2 months for a nitrate sensor [13]. The cause of this superior signal stability could lie in the PVC membrane, which confers specificity to the ISE, and also effectively shields the LIG surface from detrimental adsorption of contaminants.

## Morphology variation and effect on sensor performance

LIG is not a strictly defined material. Instead, the term “laser-induced graphene” describes a multitude of similar porous carbons which are broadly grouped by their common fabrication method (laser graphitization) and similarities in their Raman spectra. Due to differences in process input-parameters (i.e., laser wavelength, delivered energy density, applied power density, step interval, number of repetitions on same location, gas environment, source material), the resulting LIG displays differences in the following output-parameters: microstructure/morphology, specific surface area, pore-size distribution, degree of crystallinity, elemental composition, and surface chemistry. These differences in material characteristics will affect the outcome of electrochemical measurements via the degree of aqueous wettability, molecular adsorption behavior, density of states/electron transfer resistance, and bulk electrical conductivity.

In an early study, Tilakos and coworkers, who wrote a concise description of PI graphitization by laser, varied the power, scan speed, and step interval over the available operating window and identified five different morphic groups of LIG [40]. These were extensively characterized physically and spectroscopically without focusing on any particular application. The authors briefly mentioned that superhydrophilic LIG would be suitable for sensor applications. Abduhafez et al. elegantly investigated the influence of power and focal distance of the laser beam when creating a single line of LIG, and visually identified three morphologies: porous formations, cellular networks, and wooly fibers [17]. Using a slanted support, all morphologies were created on the same line of LIG, which helped locating the exact positions of morphic transitions. The authors comprehensibly linked these transition points to fixed values of laser fluence independent of laser power. As laser fluence neatly bundles several input-parameters, it was identified as primary factor in several reports. However, fluence alone cannot fully describe the conversion process, because the time dimension has been eliminated, which dictates temperature buildup in the source material. A more rigorous model to predict LIG morphologies would need to work with temperatures and temperature–time profiles, as these are at the core of the underlying physicochemical transformations. Aside from the versatility of the LIG process, seeing different groups report different sets of morphologies should not surprise. After all, microscopic classification is inherently subjective and technical differences among available laser systems likely also influence the outcome. One often observed morphology is laser-induced graphene fibers (LIGF). These structures that can grow into millimeter-long tendrils exhibited higher areal capacitance than flat types of LIG and were first created by Duy et al. by minimizing beam overlap [15].

Since wettability critically influences the area of the electrode–electrolyte interface, it warrants further attention in a discussion on chemical sensors. Tour’s group demonstrated early how scribing in an atmosphere of inert gas resulted in hydrophobic LIG [14], and a recent publication showed how to conveniently tune wettability by double lasing [13]. This report by Claussen’s group contains cyclic voltammograms of the [Fe(CN)_6_]^3−^ probe on different LIG morphologies. Interestingly, the shape of voltammograms was influenced mostly by sheet resistance and less by wettability, e.g., two electrodes with equal presenting CVs had the same resistance of around 15 Ω sq^−1^ but very different contact angles of 20° and 75°. SEM showed that the electrochemically best performing surfaces were rather flat. However, this report in combination with others, e.g., Li et al. [14], shows that one cannot infer wettability from the shape of structures in SEM images alone: two different LIG forests that look similar in SEM may differ strongly in wettability, depending on surface chemistry and adsorbed contaminants, as mentioned in the “Introduction” section. While this study focused on a second lasing step performed onto already formed LIG, it would be useful to have the same information (sheet resistance, contact angle, voltammograms with different redox probes) for different morphologies created during initial laser scribing.

Our lab published a small study on the correlation between LIG fabrication settings and voltammetric performance, which was limited to the [Fe(CN)_6_]^3−/4−^ redox probe and also paid no particular attention to the microstructures themselves [16]. However, we implicitly followed the uncontroversial assumption that electrochemical sensors benefit from high wettability. However, the lab of Feng Xinjian is experienced with hydrophobic electrodes and recently created an enzyme sensor based on superhydrophobic LIG [41]. Higher oxygen supply from air pockets inside the hydrophobic LIG resulted in a 20 times higher reaction rate of the oxidase enzyme and 60 times wider linear detection range of their H_2_O_2_ sensor. Incidentally, the authors did not adjust laser fabrication parameters to achieve hydrophobicity but used a PDMS coating instead.

In summary, while there are many reports on the influence of various production parameters on various material characteristics of LIG, fewer reports discuss the influence on electroanalytical performance. However, generalization about electron transfer rates is not possible since those depend on interactions of the given electroactive species with the electrode surface chemistry. Still, one can infer from available reports how to increase the amount of oxygen-containing groups in LIG and how to minimize sheet resistance. Therefore, overall, we think that the importance of the primary LIG morphology in most cases likely retreats behind the effect of further surface modifications necessary for chemical sensing or biosensing.

## Outlook

Statements in the literature about the shelf-life or long-term signal stability of LIG electrodes are seemingly rare and often contradictory. Due to the chemical and structural variability of the LIG material and multitude of electrochemically detected species, this is not surprising. The following trends can be concluded based on our own data and literature reports. Unmodified LIG electrodes are affected by adsorption of environmentally present organic contaminants as to be expected by a porous carbonaceous surface. This adsorption dramatically influences the detection analytes that undergo inner-sphere type electron transfer, whereas it has little to no effect on outer-sphere redox species, although voltammograms will in both cases be affected by lowering the double-layer capacitance over time. Application of a thin protective film, e.g., PVC, PVA, or chitosan (as published by Wen’s group [20]), seems a sound strategy for long-term electrode storage, to avoid hydrocarbon contamination and preserve hydrophilicity. While many LIG sensors in literature are reported to have a relatively good shelf-life, this may be connected to incidental protection through the layers of surface modifications necessary for the mechanism of the sensor.

Many academic workgroups have started using the LIG method in their research which is a good basis for the maturation of the technique and the coming years will likely see many more descriptions of sophisticated LIG-based biosensors—see Lahcen et al. for a recent review [2]—and some more studies on process-property relationships. After almost 10 years since the first report on simple LIG fabrication and several granted patents, the expected commercial success of LIG electrodes for point-of-care sensing remains to be seen. We take this as a hint that a little more effort is needed toward improving shelf-life and reproducibility. Still, LIG electrodes have already taken the first challenge and demonstrated to be highly attractive for low-cost electrochemical testing. LIG can become an affordable platform technology to meet a community’s sensing needs in a reliable, sustainable, and emancipatory way. Instructions for inexpensive fabrication and measuring equipment are already published [18], as well as methods to substitute polyimide with paper [4]. Establishing best practices on how to reliably produce, modify, and keep those electrodes sounds like a manageable task for the near future.

### Supplementary Information

Below is the link to the electronic supplementary material.

Supplementary file1 (PDF 1568 KB)

## Data Availability

The datasets generated during and/or analyzed during the current study are available from the corresponding author on reasonable request.
