# Supplementary material for: Laser-induced graphene trending in biosensors: understanding electrode shelf-life of this highly porous material
Source: Anal Bioanal Chem. 2023 Dec 12;416(9):2097–106. doi: 10.1007/s00216-023-05082-y (PMC10950954; doi:10.1007/s00216-023-05082-y)
Supplement: Supplementary file 1 — Supplementary file1 (PDF 1568 KB) [file 216_2023_5082_MOESM1_ESM.pdf]

Online Resources (Supplementary Information)

Laser-induced graphene trending in biosensors: understanding electrode shelf-life of this highly porous material

Analytical and Bioanalytical Chemistry

Authors: Arne Behrent, Veronika Borggraefe, Antje J. Baeumner*

Affiliation (all authors): Institute of Analytical Chemistry, Chemo- and Biosensors, University of Regensburg, Universitätsstraße 31, 93053 Regensburg, Germany

Email: antje.baeumner@ur.de

Experimental methods in reference to chapter 2

Reagents

All water used in this study for the preparation of any solutions and for contact angle measurements was of purity grade 1 obtained from a Millipore water system (resistivity > 18 MΩ cm @ 25 °C, TOC < 10 ppb). All chemicals were obtained in analytical grade and used without further purification.

Sample fabrication and storage

LIG electrodes (see photo in Fig. S 1A) were prepared with a flatbed CO_2 laser cutter machine (VLS2.30 with P_{max} = 30 W, λ = 10.6 µm, Universal Laser Systems Inc., USA) using polyimide sheets (Kapton HN500, 125 µm thickness, DuPont, USA) as substrate which were placed in focus of a 125 µm diameter beam ($1/e^2$). Substrates were cut into pieces of suitable size and secured in place with masking tape to avoid movement and rippling. The electrode design in Fig. S 1B was used for voltammetry and, as seen in the sketch, active electrode area, current collectors and contacts to socket all consisted of LIG. For contact angle measurement, an array of six squares with 1 cm length was used, as depicted in Fig. S 1C. The machine settings were 1% power, 10% speed and 1000 by 1000 pulses per inch, unless stated otherwise.

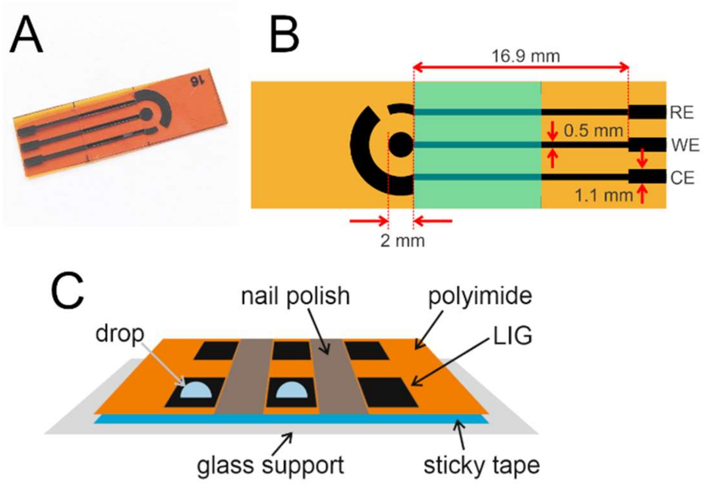

Fig. S 1:Photo (A) and sketch (B) of electrode for voltammetric measurements and perspective on sketch of contact angle measurement (C)

Immediately after fabrication, samples were stored in one of the following ways (see Fig. S 2): on a small paper shelf above a lab bench, in a segmented, non airtight, polypropylene (PP) box (Hünersdorff GmbH, Ludwigsburg, Germany), fixed with tape on a paper sheet inside a drawer, or inside round-bottom flasks made of borosilicate glass. The flasks were previously cleaned thoroughly with acetone and glass stoppers were fixed without grease. Wide-necked flasks were used to allow for insertion of the samples without bending. All samples were kept in a non-air conditioned chemical laboratory at temperatures between 15 and 28 °C.

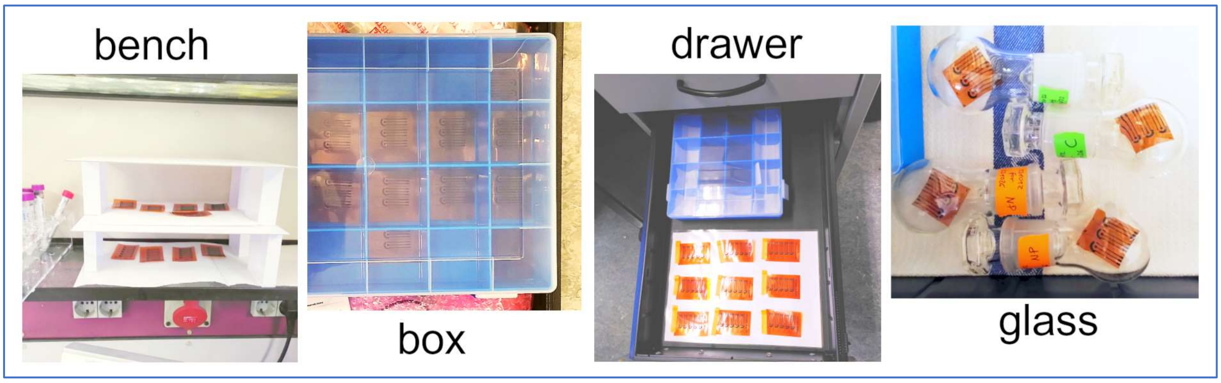

Fig. S 2: Photos of LIG samples stored in different locations/containers

In order to electrically insulate current collectors and to confine the size of the electrode area, commercial transparent nail-polish was applied manually (see green area in Fig. S 1B). In comparison to adhesive tape as delimiter, nail-polish was found more resistant to leaking and more convenient to apply. However, the nail-polish is also a likely source of hydrocarbon contaminants which may become significant over long storage times. When comparing storage environments, electrodes were kept without nail-polish and only insulated after being taken out of storage, while allowing 35 min of drying time before measurement.

When the presence of nail-polish was investigated as a factor, glass flasks were used exclusively as storage containers and nail-polish was let to dry for 35 min under ambient conditions before samples were put in. Here, all flasks with samples were kept at a constant 21 °C in a dark cabinet. Over the course of experiments, the initially chosen commercial nail-polish (np#1) suddenly became unavailable, forcing us to switch to another seemingly similar brand (np#2). Later investigation showed that the choice of nail-polish brand had strongly influenced capacitance over storage time. A list of components of np#1 and np#2 can be found in Tab. S 1.

Measurements

Static water contact angles (WCA) were measured optically via the sessile drop method on an OCA 25 system with corresponding software (DataPhysics Instruments GmbH, Filderstadt, Germany). No environmental chamber was used. A volume of 3 µL DI water was suspended from an automatic syringe, then transferred to the sample by vertical movement of the stage, followed by quick acquisition of an image series. To handle LIG samples, they were fixed onto glass slides with double sided adhesive tape and measurements were done timely after removal from storage. LIG is very porous and the contact angles on a hypothetical flat surface of the same chemistry would probably differ from the ones observed here, which are not suitable for further calculations. However, the data obviously still allow conclusions about wettability and can be used to directly compare between samples of a similar porous nature.

For all electrochemical measurements, LIG electrodes were horizontally plugged into a three-contact socket which was fastened to a movable stage and connected to a PalmSens4 potentiostat (PalmSens BV, Netherlands). The material of the LIG contact areas was found robust enough for reproducible electrical connection even when repeatedly re-plugging them into this particular sliding socket, that modification with e.g. silver paste or sticky copper tape was not found necessary and omitted. In every measurement, a priming volume of 50 µL was pipetted center onto the WE, then gently removed with a paper wick and replaced with another 50 µL of the same solution for measurement. The stage was then lifted to contact the droplet with a positionally fixed Ag/AgCl reference electrode (3 M KCl, BASinc., USA), while the LIG pattern designated as “RE” was not used.

After measurement, LIG electrodes were pulled from the socket and discarded. A photo of the electrochemical setup is provided in Fig. S 3.

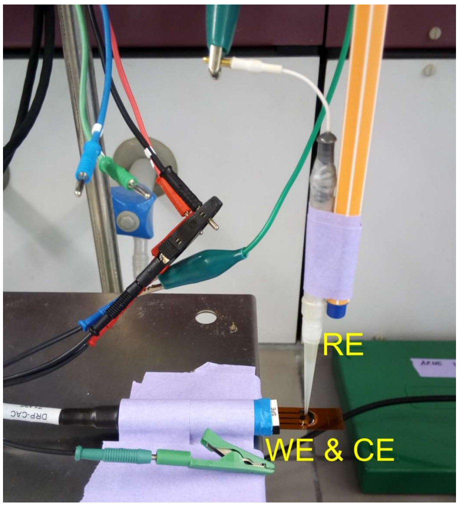

Fig. S 3: Photo of electrochemical cell

The capacitance was determined by cyclic voltammetry (CV) in phosphate buffered saline (PBS) of pH 7.4 (8.1 mM Na_2HPO_4, 1.9 mM KH_2PO_4, 2.7 mM KCl, 137 mM NaCl). Cyclic voltammograms were collected at different scan-rates (v) between 10 and 400 mV s-1 in a 100 mV-wide potential window centered on the open-circuit potential, the value of which was estimated from the endpoint of a 30 s measurement. The capacitive current (I_C) of each directional scan was averaged from the roughly stable second half and 10 s pauses were kept between scans. The slope of I_C vs v yields the averaged capacitance of the electrode according to $C = \frac{I_C}{v}$ (see Fig. S 4 for representative data).

Since the construction of (bio)analytical sensors is our main use-case for LIG electrodes, we were interested in the change in signal from faradaic electrode reactions vs electrode storage-time. As redox probes, we chose potassium(III)hexacyanoferrate ($K_3[Fe(CN)_6]$), hexammineruthenium(III)chloride ($[Ru(NH_3)_6]Cl_3$) and acetaminophen to cover different modes of charge transfer. Stock solutions were prepared in PBS at 10 mM concentration and kept in the fridge at 4 °C in falcon tubes over the course of experiments, a maximum time of 3 months. Working solutions of 1 mM were prepared on each day of measurement by removing a portion of stock, letting it assume ambient temperature and mixing 100 µL stock with 900 µL PBS in PP tubes, followed by mixing and repeated once more for 0.1 mM solutions. A pair of two consecutive CVs were recorded at 50 mV s^{-1} and 1 mV step, starting in cathodic direction for $K_3[Fe(CN)_6]$ and $[Ru(NH_3)]Cl_3$ and starting in anodic direction for acetaminophen. Each electrode was used for only one measurement and then discarded.

Data evaluation

Figures were created with R/ggplot2. The discussion of Fig. 1 and Fig. 2 in the manuscript mentions the differences in magnitude between the means of the earliest and latest dataset of each group. Double-sided unequal variances t-tests were calculated with R and reported in the form of "t(DF)=T-STATISTIC, P-VALUE".

Additional results

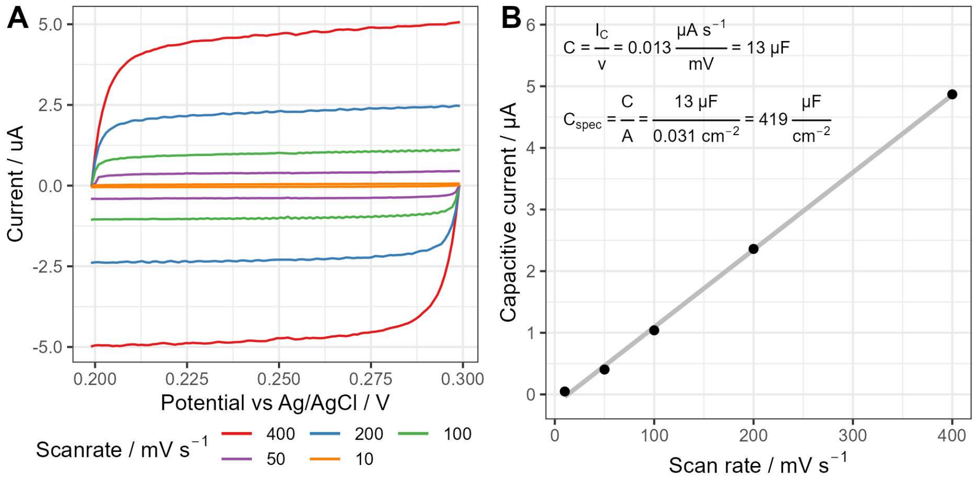

Fig. S 4: Representative voltammograms recorded on one LIG electrode in PBS (A) and plot of capacitive current vs scan-rate with calculation of area-specific capacitance (B)

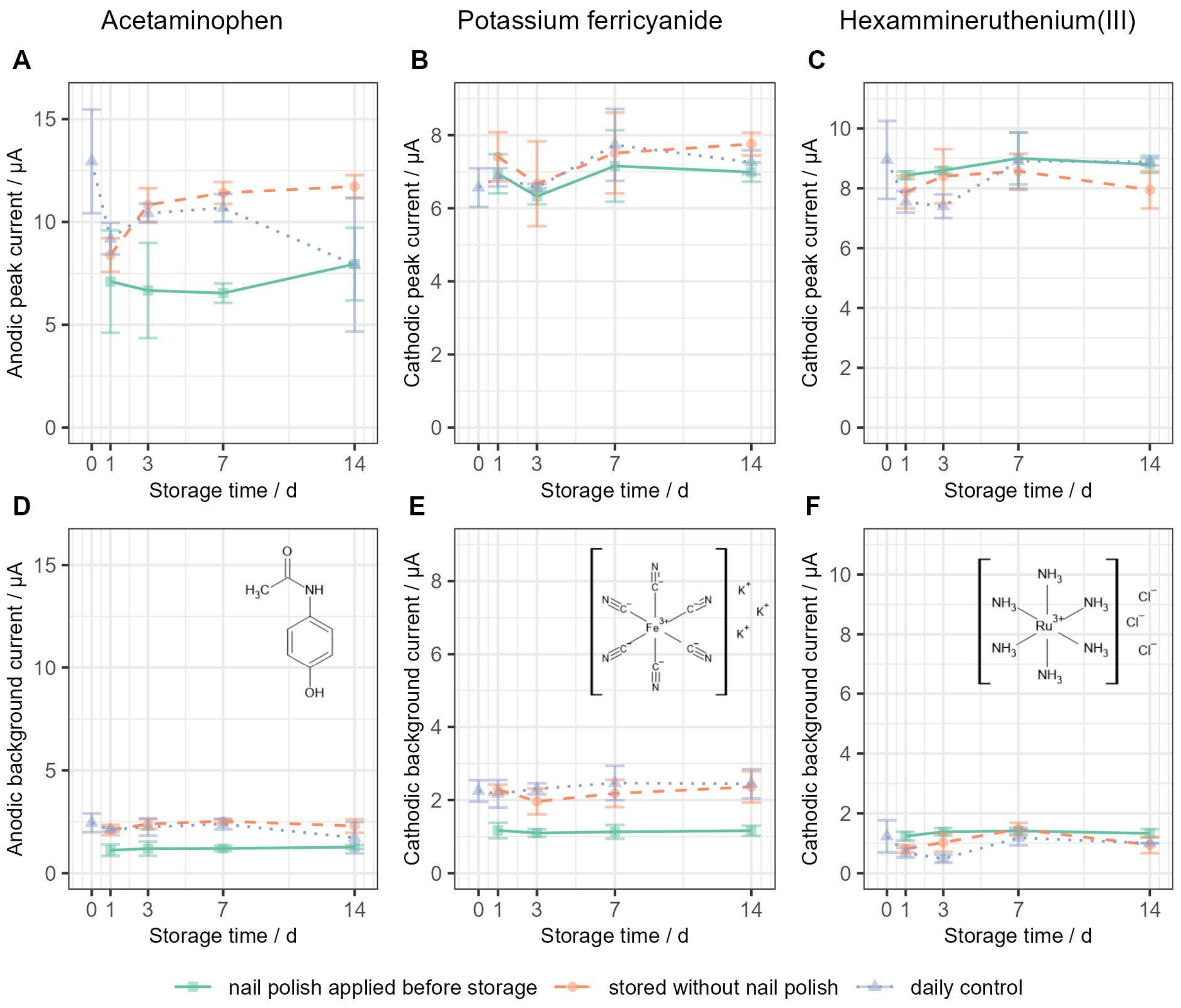

1

Fig. S 5: Peak currents and background currents in CV recorded with 0.1 mM acetaminophen (A, D), 1 mM ferricyanide (B, E) and 1 mM hexammine-ruthenium(III) (C, F) in PBS buffer over the course of 14 days. Electrodes were stored in glass flasks with or without nail-polish (nail-polish brand "Catrice" (see Tab. S 1) was used). Points and error bars represent mean and 1X standard deviation of the sample.

Tab. S 1: Components of nail-polishes used in the study according to store website (www.mueller.de) – shared components are colored grey

np#1 ("essence 2in1 base & top coat", Prod.ID="648048")			*np#2* ("Catrice Natural All in 1 Hardening Base &Top Coat", Prod.ID="2654210")		
Rank	ingredient (INCI name)	function	Rank	ingredient (INCI name)	function
1	ETHYL ACETATE	solvent	1	ETHYL ACETATE	solvent
2	BUTYL ACETATE	solvent	2	BUTYL ACETATE	solvent
6	ISOPROPYL ALCOHOL	solvent	9	ISOPROPYL ALCOHOL	solvent
			5	ALCOHOL	solvent
4	NITROCELLULOSE	film former	3	NITROCELLULOSE	film former
3	PHTHALIC ANHYDRIDE / TRIMELLITIC ANHYDRIDE/GLYCOLS COPOLYMER	film former	6	POLYESTER-23	film former
7	ACRYLATES COPOLYMER	film former	10	TRIETHOXYCAPRYLYLSILANE	film former
5	ACETYL TRIBUTYL CITRATE	plasticizer	4	TRIETHYL CITRATE	plasticizer, fragrance
8	CI 60725 (VIOLET 2)	colorant, synthetic	8	ALOE BARBADENSIS LEAF EXTRACT	skin conditioning
			11	CI 77007 (ULTRAMARINES)	colorant, inorganic
			7	CARTHAMUS TINCTORIUS (SAFFLOWER) SEED OIL	skin conditioning
